# The impact of intranasal oxytocin administration and social observation on time perception and task execution in a simple motor task

**DOI:** 10.1007/s00426-026-02290-w

**Published:** 2026-04-07

**Authors:** Orsolya Kiss, József Topál, Dorottya Berkes, Karolin Török-Suri, János Horváth

**Affiliations:** 1 Center for Health Sciences, SRI International, 333 Ravenswood Ave, Menlo Park, CA 94025 USA; 2 Institute of Cognitive Neuroscience and Psychology, HUN-REN Research Centre for Natural Sciences, Magyar tudósok körútja 2., 1117, Budapest, Hungary; 3 ELTE-HUN-REN NAP Comparative Ethology Research Group, HUN-REN Research Centre for Natural Sciences, Magyar tudósok körútja 2., 1117, Budapest, Hungary; 4 Department of Cognitive Science, Faculty of Natural Sciences, Budapest University of Technology and Economics, Műegyetem rkp. 3., 1111, Budapest, Hungary; 5 Institute of Psychology, Károli Gáspár University of the Reformed Church in Hungary, Bécsi út 324, 1037, Budapest, Hungary

## Abstract

Most everyday tasks allow for considerable freedom in task performance. Besides taking physical constraints into account, we also rely on social cues to ascertain that our performance is acceptable. The presence of an observer influences performance, and oxytocin, a neuropeptide, enhances social sensitivity. This study examined the modulatory effects of intranasal oxytocin administration and the audience effect on motor adaptation and time estimation performance in a simple timing task. 18 adult male participants performed a time-interval production task using a force-sensitive device under oxytocin versus placebo, with or without an observer. The results showed that participants produced longer time intervals when oxytocin was administered, while the presence of an observer led to a shortening of the time intervals and a decrease in applied force indicating altered motor planning. Notably, under observation, the effects of oxytocin were enhanced, manifested as a further reduction of planned force application. Motor adaptation (the reduction of applied force) was more marked with successive repetitions, and with the presence of an auditory action-effect. Anxiety levels were positively correlated with the prolongation of timing intervals and force increase, aligning with the literature on anxiety’s impact on motor performance. This shows that oxytocin and social observation independently and interactively affect time perception and motor performance, thus demonstrating oxytocin’s influence on cognitive processes beyond purely social behaviors. The present study also demonstrates how force application patterns may serve as readouts of social cue utilization behavior in simple everyday tasks.

**Supplementary Information:**

The online version contains supplementary material available at 10.1007/s00426-026-02290-w.

## Introduction

Social sensitivity plays a fundamental role in shaping human behavior, particularly in contexts where actions are performed in the presence of others. Beyond explicit social interaction, merely being observed can alter performance across a wide range of motor, cognitive, and affective domains - a phenomenon widely described as the audience effect (Guerin, 1986). Awareness of observation can modulate arousal, motivation, and behavioral strategies, resulting in either facilitation or impairment depending on task demands and individual state (Wallace et al., 2005; Belletier et al., 2015). Such effects are especially relevant in experimental settings, where participants may implicitly adjust their behavior in response to perceived evaluation or demand characteristics (Orne, 2009, 2017). Oxytocin, a neuropeptide produced in the hypothalamus, has been shown to enhance social sensitivity (Kramer et al., 2006) and bonding, suggesting that it plays a significant role in modulating social behaviors and emotional responses (Kis et al., 2013). The influence of the oxytocinergic system on individual responses to social stimuli, including cues related to social attention such as gaze and eye contact (Soriano et al., 2020; Guastella et al., 2008), suggests a fundamental connection between neurochemical processes and social cognition.

Humans are also inherently sensitive to the visual attention directed towards them, commonly referred to as the ‘audience effect’ (Guerin, 1986). This term in social psychology describes how an individual’s behavior may change when they are aware of being observed. The presence of an audience can influence a range of activities, from prosocial behavior (Guala & Mittone, 2010), sport performance (Böheim et al., 2019), to complex cognitive operations (Lombardo & Catalano, 1975; Belletier et al., 2015), including video gaming (Bowman et al., 2013), with the potential to both enhance (Burnham & Hare, 2007; Fullwood & Doherty-Sneddon, 2006; Bowman et al., 2013) and impair it (Belletier et al., 2015). Importantly, the audience effect may also contribute to how participants behave in social-experimental situations, that is, whether and how they comply with genuine or perceived demand characteristics (Orne, 2009, 2017). On one hand, an audience can provide a stimulus that elevates arousal and enhances performance through increased focus and motivation (Cañigueral & Hamilton, 2019). On the other hand, it can induce performance anxiety, resulting in decreased efficiency and accuracy (Wallace et al., 2005). It is also reasonable to assume that oxytocin, with its widely reported role in social cognition (for a review see Ebert and Brüne, 2018), may modulate the audience effect, impacting an individual’s performance and social engagement. As a neuropeptide associated with social bonding and the reduction of social anxieties (Neumann & Slattery, 2016), oxytocin could potentially attenuate the stress-related aspects of the audience effect, thereby allowing the performance-enhancing elements to prevail. Conversely, by increasing the salience of social cues (Shamay-Tsoory & Abu-Akel, 2016), oxytocin might also intensify the individual’s awareness of being observed, which could enhance performance anxiety in some contexts. This interaction may well play a substantial role in social anxiety, where being the focus of attention can dramatically affect one’s response. Despite these insights, the specific effects of oxytocin on task performance within a social context remain largely unexplored, and investigating the interaction between oxytocin and the audience effect on performance can help to elucidate the neurochemical underpinnings of social facilitation and inhibition. Understanding the modulating role of oxytocin in this dynamic could illuminate aspects of social cognition and provide insights into the neurobiological aspects of social behavior.

Taken together, these findings suggest a theoretical link between oxytocin, social observation, and motor adaptation. Oxytocin enhances the salience of social cues and modulates arousal, while the presence of an observer can alter motor execution through social facilitation or inhibition. Motor adaptation tasks, particularly those using force-sensitive measures, provide a sensitive behavioral readout of these influences, as they capture both planned motor output and online adjustments. From this perspective, oxytocin may amplify the audience effect by heightening sensitivity to observation, thereby influencing how participants adapt their motor actions and perceive time. This framework motivates our investigation of oxytocin × observation interactions in a simple motor timing paradigm.

In the present experiment, we utilized a simple motor timing task to investigate interactions between participants’ susceptibility to the audience effect, as operationalized through bystander observation, and intranasal oxytocin administration. Participants were required to briefly press a force sensitive resistor (FSR) every 4 s on their own (i.e., without external cues), without lifting their finger from the device between presses. Previous studies administering such tasks showed that the considerable freedom in action execution (i.e. the device can be successfully operated with a broad range of force) gives rise to motor adaptation patterns that allow insight into participants’ task representations when the sensory consequences of the actions are manipulated (Horváth et al., 2018; Neszmélyi & Horváth, 2018; Varga et al., 2022). The FSR, in contrast with a typical button, provides no distinct transient tactile or auditory feedback (click) when the action is registered, therefore, it is less obvious whether the action succeeded or not. This uncertainty is reduced when the actions consistently elicit a computer-generated sound, and the consistent presence and absence of such an auditory feedback affects participants motor output: in the absence of feedback they exert more pressure to ensure that the action was registered by the device (Neszmélyi & Horváth, 2018; Horváth et al., 2018; Horváth, 2024).

The presence of an experimenter watching task execution may influence motor output in several ways. Because operation of the device is an isometric action (that is, the finger and arm do not move during in the task) participants may apply more force, to create visually more conspicuous signs of muscle tension, and thus visually demonstrate their compliance with the instructions – that is, participants may exaggerate the action to provide a readily observable non-verbal signal for the experimenter. But they may also apply less force because the absence of corrective action by the experimenter reassures them that the on-going task execution satisfies task criteria – and such an effect might be more pronounced in the absence of sound feedback, when the participant’s uncertainty about the success of the interaction is high. In sum, we hypothesized that social stimulation would be reflected in the FSR task execution, and such effects would be amplified by the administration of oxytocin.

Timing performance may also be influenced by the presence of a human observer. Time perception, a cognitive function essential to human behavior, is not exempt from the influences of social context (Bak, 2022). The perception of social interaction can compress subjective duration (Liu et al., 2018), with oxytocin playing a role in mediating this effect (Liu et al., 2018; Colonnello et al., 2016). Liu et al. demonstrated that social interactions tend to be perceived as shorter in duration compared to interactions where agents act non-communicatively (Liu et al., 2018). They also showed that intranasal oxytocin administration restores the temporal compression effect in socially less proficient individuals, whereas the administration of an antagonist of oxytocin diminishes the effect in socially proficient individuals. One explanation could be that oxytocin enhances the salience of social stimuli (Froemke & Young, 2021), especially if the stimuli is personally relevant to the beholder (Colonnello et al., 2016). In line with this, it has been reported that the processing of self-relevant information affects time perception, with higher self-consciousness leading to an elongation of subjective time and lower self-awareness causing it to contract (Wittmann, 2015). Social stress has also been reported to lead to the overestimation of time durations (Bagley et al., 2021; Van Hedger et al., 2017). The “socio-temporal brain,” proposed as a conceptual framework describing neural systems that integrate social and temporal processing (Schirmer et al., 2016), is suggested as the core of these effects. This framework posits that social interaction and time perception rely on partially overlapping neural mechanisms, supported by widely distributed networks involving dopamine, oxytocin, and serotonin systems (Liu et al., 2018), and the interaction between oxytocinergic and dopaminergic pathways (Love, 2014). With respect to oxytocin, our study aims to investigate how oxytocin administration impacts time perception in the neurotypical population. We expect that if oxytocin indeed enhances sensitivity to social stimuli, oxytocin administration would likely cause individuals to perceive time as passing more quickly, especially in socially intense audience conditions. However, if oxytocin enhances the individuals’ inner motivation systems, we expect to see an effect aligned with the participants’ overall emotional state.

Given that stimulus-filled intervals are perceived as being longer than empty intervals (Thomas & Brown, 1974), auditory feedback provided by one’s own actions may also result in the overestimation of the time passed since the last action, and thus result in shorter produced intervals. Indeed, three out of five studies using similar paradigms reported such an effect (Neszmélyi & Horváth, 2017; Neszmélyi et al., 2022; Horváth, 2024; the other two reported null-effects: Horváth et al., 2018; Neszmélyi & Horváth, 2019).

Overall, our study aims to gain a better understanding of the impact of intranasal oxytocin and the presence of an audience, a social stimulus on time perception and task execution. This task incorporates the manipulation of a simple sensor, the option of auditory feedback, and subjective time estimation; components, that frequently co-occur in real-life situations. To our knowledge, this is the first study to experimentally examine how oxytocin administration and social observation jointly influence timing and motor adaptation in a simple task.

## Methods

### Participants

Twenty-one adult male volunteers between the ages of 20 and 50 participated in the study. Out of the initial sample of 21, three individuals were excluded due to missing data, which resulted from technical problems. The mean age of the remaining sample was 26.33 years, SD: 7.15. Participants were recruited from Budapest, Hungary through advertisements at local universities and on various online pages. Participants were instructed to abstain from food, drink (other than water), alcohol, smoking and caffeine for three hours before the in-person visits. The study protocol was approved by the United Ethical Review Committee for Research in Psychology (Ref. No. EPKEB 2015/23). The study was conducted according to the Declaration of Helsinki. All participants gave written, informed consent before participation.

Previous studies showed that in similar tasks the effect of adding or removing a tone action-effect on motor adaptation is extremely robust, with r-effect size estimates above 0.73 across numerous studies (Volosin et al., 2025; Neszmélyi & Horváth, 2017, 2018; Horváth, 2024). The power to detect such an effect with an alpha level of 0.05 in the present sample final size of 18 was 0.96 (determined by the *pwr* package, version 1.3-0, Champely 2006). We are, however, unaware of any previous experiments investigating the effects of the presence of an audience, or intranasal oxytocin administration on similar force application patterns or timing behavior, thus these aspects of the present study should be regarded as explorative.

### Procedure

All participants completed online screenings to get informed about the study and to assess their general eligibility. Participants were invited for two in-person visits with at least three days, but not more than two weeks between the first and second visit. Upon arrival, participants completed an informed consent form. Before the administration of the oxytocin or placebo, the participants were asked to fill in the State-Trait Anxiety Inventory (STAI) questionnaire (Spielberger et al., 1983).

#### Substance administration

We used a double-blind within-subject design, where the participants’ responses were investigated in a ‘Motor task’ (see below) during both visits: once after receiving intranasal oxytocin (24 IU oxytocin, 3 puffs per nostril, Syntocinon, Novartis) and once after receiving a placebo (isotonic sodium chloride 0.9% solution, 3 puffs per nostril) treatment. The order of the two treatments was randomized between visits. The oxytocin or placebo administration was followed by a 35-minute-long waiting period, consistent with pharmacokinetic guidelines for peak oxytocin activity (Guastella et al., 2013). Participants were instructed to wait without using any electronic devices (and asked to turn off their mobile phones). During this waiting period, they were offered magazines with no human figures or images (e.g. car magazines), and left alone quietly sitting in an isolated room. After the waiting period, participants completed an arithmetic task for approximately 30 min, followed by the motor task. Participants completed an arithmetic task as part of a broader research program; analyses of this task will be reported elsewhere.

#### Motor task

Participants were seated in a quiet room at a table, facing either an empty chair (no audience present) or the experimenter (audience present), who was facing a computer screen. The experimenter and the participant sat on opposite sides of a table. The experimenter was a female researcher in her mid-twenties. When present, the experimenter followed standardized, scripted instructions and did not engage in task-irrelevant conversation.

**Fig. 1:**
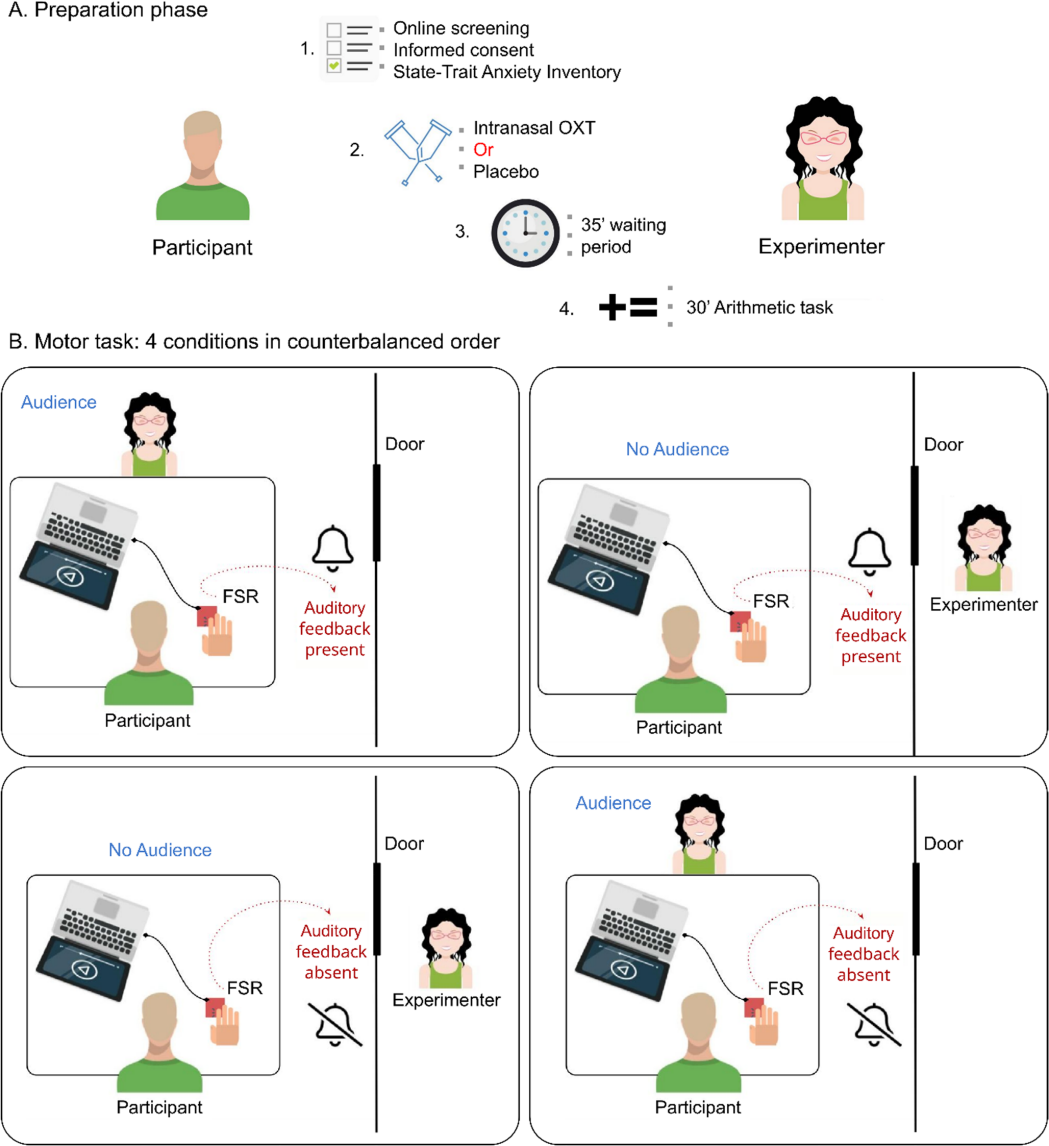
Overview of the sequential phases of the study; beginning with the preparation phase followed by the execution of the motor task in four different conditions

Participants performed a timing task: they rested the forearm of their dominant hand (*N* = 1 left-handed, *N* = 17 right-handed) on the table, and were instructed to keep their index finger on a paper-thin force sensitive resistor (FSR, Model 406, Interlink Electronics, Westlake Village, CA, USA – 0.3 mm thick, with a square, 39.3 mm x 39.3 mm active area) - taped on the table in front of them, and press it briefly every four seconds, without lifting their finger from the FSR between presses as demonstrated by the experimenter. Before the first press participants were asked to count to 10. Each condition lasted three minutes. The FSR signal was sampled at a rate of 658 Hz by an Arduino UNO (Arduino s.r.l., Monza, Italy) microcontroller board. A press onset was registered when the applied force exceeded a threshold of 1.48 N after being continuously under the threshold for at least 10 ms (and press offset was identified as the first timepoint following the onset after which force remained continuously under the threshold for at least 10 ms). In some conditions (see below), press onsets elicited a well-audible, but comfortable-level auditory effect through a loudspeaker: a 1-ms-long sound impulse – a click sound. Participants were instructed to keep their eyes open, time their presses independently, and continue the task until signaled by the experimenter. This procedure was repeated in four conditions constituting all possible (2 × 2) combinations of two factors: the presence of the experimenter (audience vs. no audience) and the presence of auditory feedback (with auditory feedback vs. without auditory feedback), in a counterbalanced order. When the experimenter was present in the room, she observed the motor task while facing the participant, alternating between extended gazes at the participant’s face and brief glances at the screen and sensor. Eye contact was not experimentally manipulated but formed part of the naturalistic observation context intended to operationalize social presence. No deliberate facial expressions, evaluative comments, or additional verbal or non-verbal cues were provided beyond the standardized procedure. The experimenter maintained a neutral demeanor across sessions. Between conditions, participants were instructed to stretch their hands to mitigate the transfer of a potential force application set between conditions. Tests were video recorded from four different angles using cameras fixed to the walls (See Fig. 1. for an overview of the study procedures.)

After the timing task, participants completed the State-Anxiety subscale of the STAI questionnaire.

### Preprocessing and measures and measures

#### Motor metrics – outcome measures

Data was preprocessed in Python 3.0. We excluded actions that strongly deviated from the target between-action interval. That is, we excluded actions that followed the previous action in less than 1 s, as small force fluctuations around the force threshold during the release of force application were sometimes misregistered as closely repeated presses (0.3% of the trials). Actions following the previous action in more than 6 s were excluded as well (6% of the trials). Because presses were only registered if the applied force exceeded a predefined threshold, intervals longer than 6 s may reflect presses that did not reach the force threshold and thus remained unregistered, or actions omitted due to lapses of attention or off-task behavior. Given the target interval of 4 s, the 6-second threshold was selected as a conservative, task-based criterion reflecting action registration constraints rather than an assumed upper bound on perceptual time expansion. Sensitivity analyses using more liberal upper limits (7000 ms, 8000 ms) and no upper limit yielded the same qualitative pattern of results (see Supplementary Table 9).

The following metrics were extracted:

Time interval between the actions (T-INT): This metric quantifies the average duration between consecutive actions (ms).

Total impulse: The integral of force (i.e., mechanical *impulse*) over the period between press onset and offset, that is, the total force exerted over the duration of the action. Because the duration of force application ranged from 3 to 1579 ms (Mean = 254.9 ms, SD = 194.1 ms), this measure reflects the motor program set before action execution (i.e. action planning) and potential online adjustments performed in response to the (sensory) effects elicited by the action itself (Neszmélyi & Horváth, 2018; Varga et al., 2022; Elliott et al., 2001). Initial impulse: The area under the force curve in the first 60 ms following action onset. This metric reflects the initial part of the motor program, which cannot be affected by potential reactions to the action-triggered sound feedback (see above), because 60 ms is well-below the humanly possible reaction time (Pain & Hibbs, 2007). That is, this metric is free from the potential effects of online force adjustments due to the perception of the elicited sound effect, and captures potential differences in an initial, planned part of the actions.

Peak force (PF): The maximum force exerted during each action.

Peak latency (PL): The time taken from action onset to peak force.

Duration of action (DA): Time elapsed from action onset to offset. 

#### Predictors

Substance administration: This predictor refers to the type of treatment given to participants. In one session, intranasal oxytocin (OXT) was administered, while in the other session, a placebo was given.

The presence of human observer: This factor distinguishes between two settings. The experimenter was either present in the room, visually observing the participant (Audience present) or remained outside (No Audience).

Employing auditory feedback: This predictor refers to whether a click sound was present or absent when pressing the FSR.

The State-Trait Anxiety Inventory (STAI) (Spielberger et al., 1983): It is a standardized questionnaire for detecting and measuring anxiety, consisting of 40 items, to which participants respond on a four-point Likert scale in terms of intensity (from “not at all” to “very much so”). The items are grouped into two scales, focusing on how participants feel at the moment, or in general to investigate (a) state anxiety, where anxiety is conceived as a particular experience, a feeling of insecurity or of helplessness in the face of perceived harm that can lead to either worry or flight and avoidance, and (b) trait anxiety, which is the tendency to perceive stressful situations as dangerous and threatening and to respond to various situations with different intensities. In the present study, both state and trait scales were assessed before the task performance, and again immediately after the motor task (post-task measure), but only the pre-task Scale 1, which is specific to state anxiety, was included in the downstream analyses.

### Statistical analysis

We used linear mixed models (LMMs, R package ‘lme4’, (Bates et al., 2015) to examine participants’ performance under four conditions with different social stimulations, following both oxytocin and placebo treatments. We report results for the dependent variables: Total impulse, Initial impulse and T-INT exclusively, as these variables are highly correlated with the other metrics (PF, PL, and DA, see Supplementary Table S1). All models included five main factors: *Substance administration* (oxytocin or placebo), *Observation* (audience or no audience), *Auditory feedback* (present or absent), and the *Order of visits* (first or second) and were controlled for participants’ state anxiety (*STAI-S*,* assessed before the substance administration)*. We did not control for the order of the conditions given that those were in a counterbalanced order by design. Baseline anxiety (pre-task) was incorporated into the current models, as it best reflects participants’ state prior to treatment and task engagement. Given the circadian fluctuations in time perception (Kuriyama et al., 2003), likely due to time-of-day differences in the dopaminergic system (Marinho et al., 2018), in the analysis of the T-INT we included the time of day as a control variable (divided into 4-hour intervals: <12PM, 12-4PM, >4PM). Two-way interactions (Substance administration x Observation; Substance administration x Auditory feedback; Observation x Auditory feedback) were also included in the model to determine divergence in performance across different scenarios. The model included the participant ID and the visit order as random effects. Continuous predictors were standardized and both Total impulse and the Initial impulse were log-transformed to normalize residual distributions.

Inferential statistics were based on likelihood ratio tests comparing nested models with and without each predictor, using the Chi-square distribution. We provide χ^2^ and p-values of likelihood ratio tests (threshold for statistical significance was adjusted with Bonferroni correction: α = 0.05 / (predictors * outcome measures) + 1, p values below 0.001 were considered significant). In order to interpret the interaction effects, we performed estimated marginal mean analysis (R package ‘interactions’, (Long, 2019). The complete likelihood ratio test results of the LMM models are provided in the supplementary material (Table S2). Supplementary Tables S3–S5 provide model estimates and effect sizes.

For an overview of the LMM analyses, see Supplementary Table S1.

## Results

### Initial impulse - characterizing action planning

The force exerted by the participant over the first 60 ms following action onset, the Initial impulse was reduced in trials where auditory feedback was provided (main effect of Auditory feedback: χ^2^(1) = 6615.03, *p* < 0.001) and during the second visit (main effect of Order: χ^2^(1) = 35.64, *p* < 0.001) (Fig S1A). Participants’ higher state anxiety scores were also associated with an increased Initial impulse (χ^2^(1) = 67.86, *p* < 0.001) (Fig S2A).

**Fig. 2:**
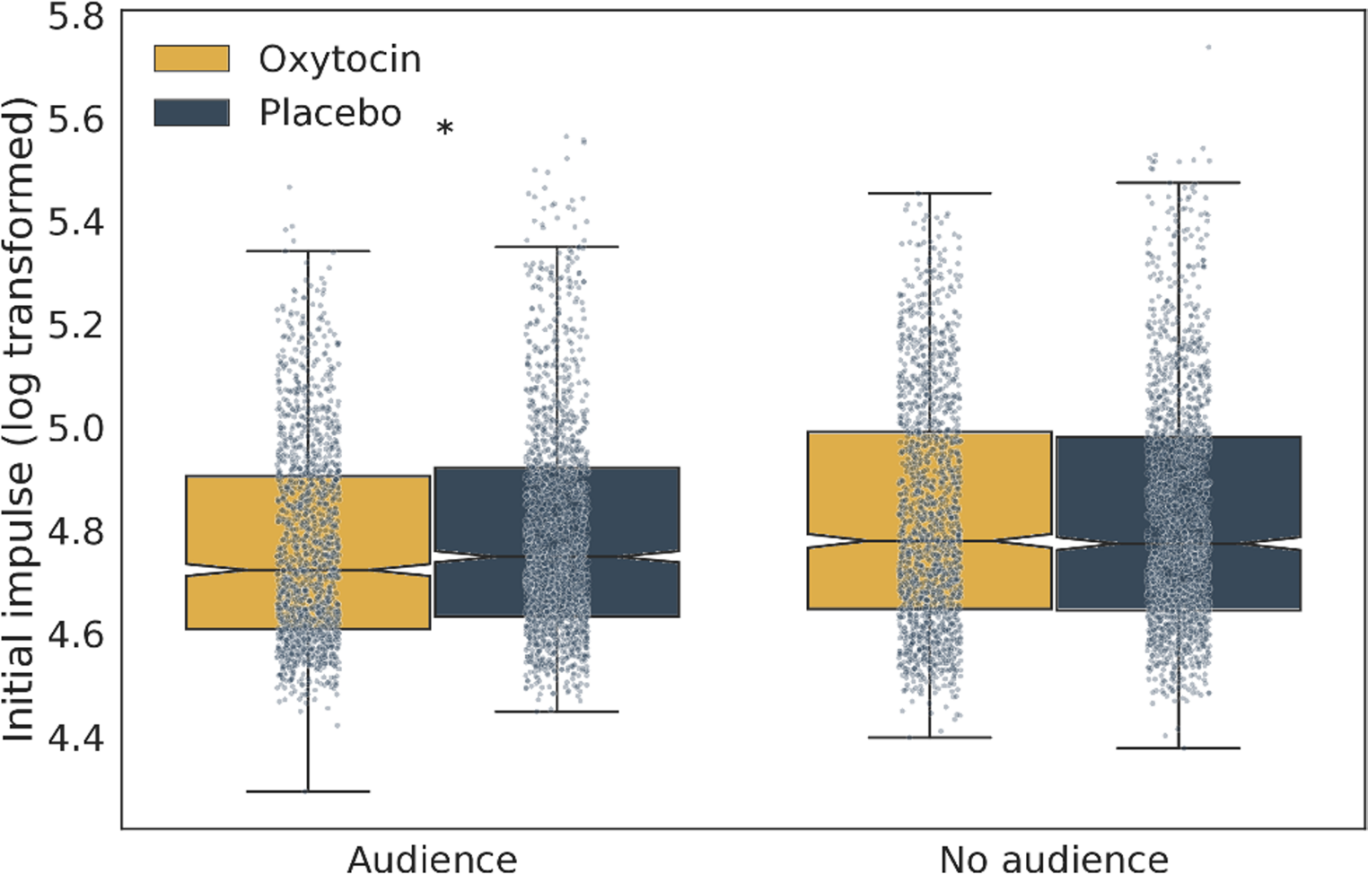
The *Observation* by *Substance administration* interaction effect on the Initial impulse, the force applied in the first 60 ms of the action, which characterizes action planning. In addition to the individual data points, the boxplots display the medians (central line), inter-quartile range (IQR, represented by the box), and outliers (individual points outside the whiskers). The whiskers cover data points up to 1.5 times the IQR from the first and third quartiles. Asterisks mark statistically significant differences. While the Initial impulse was generally higher in the absence of audience, when the experimenter observed the participants, the Initial impulse was lower under the influence of the OXT

**Fig. 3:**
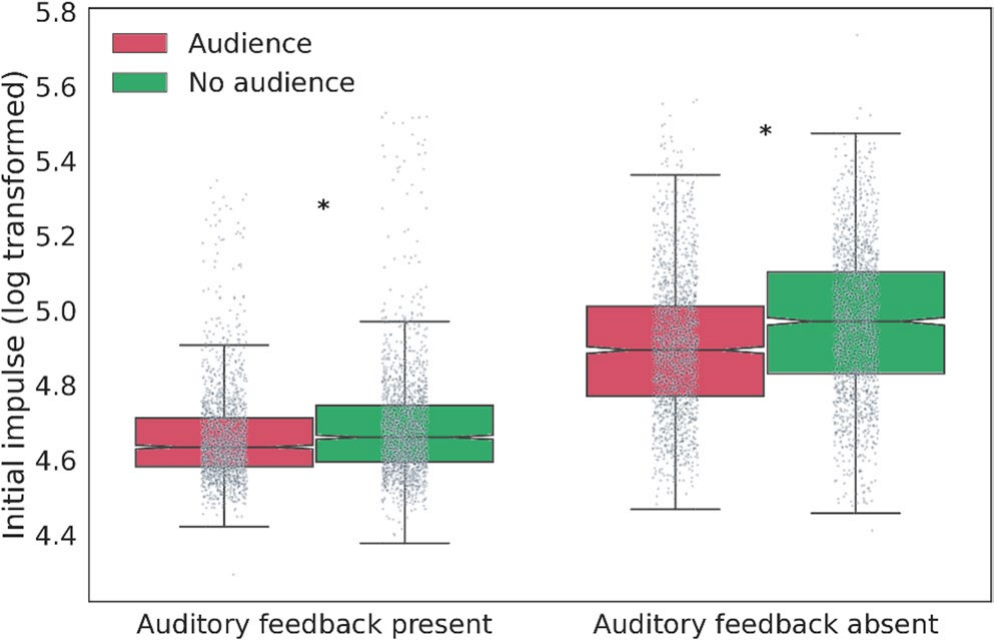
The *Auditory feedback* by *Observation* interaction effect on the Initial impulse, the force applied in the first 60 ms of the action. In addition to individual data points, the boxplots display the medians (central line), inter-quartile range (IQR, represented by the box), and outliers (individual points outside the whiskers). The whiskers cover datapoints up to 1.5 times the IQR from the first and third quartiles. Asterisks mark statistically significant differences. While the Initial impulse was generally higher without auditory feedback, it was largest when participants had neither auditory feedback nor observation. Moreover, when the experimenter observed the participants, the Initial impulse was lower during the Auditory feedback condition than when there was no audience

Moreover, both the main effects of Substance administration (χ^2^(1) = 28.49, *p* < 0.001) and Observation (χ^2^(1) = 196.01, *p* < 0.001) had a significant impact on the Initial impulse, that is, participants exerted less force when under the influence of OXT, or when being observed by a human. However, we have found significant two-way interactions between the Substance administration and Observation (χ^2^(1) = 14.06, *p* < 0.001; Fig. 2), as well as between Auditory feedback and Observation (χ^2^(1) = 41.80, *p* < 0.001; Fig. 3).

With respect to the Substance administration x Observation interaction (Fig. 2), participants exhibited a lower Initial impulse with auditory feedback when observed, as compared to when there was no observation. An analysis of the estimated marginal means revealed that in the absence of observation (No audience), there was no significant difference in this behavioral measure between the OXT and Placebo treatments. However, when participants were observed by the experimenter, the Initial impulse was notably reduced after the OXT but not after the Placebo treatment (estimate = 0.02, t = 6.43, *p* < 0.001).

Looking at the Auditory feedback x Observation interaction (Fig. 3), the highest Initial impulse values were recorded when participants neither received auditory feedback nor were observed, whereas the lowest values were recorded when both auditory feedback was given and observation took place. The pairwise comparison showed that there were significant differences between the Audience and No audience contexts regardless auditory feedback (Auditory feedback: estimate = 0.02, t = 5.46, *p* < 0.001, No auditory feedback: estimate = 0.06, t = 14.54, *p* < 0.001).

### Total impulse

**Fig. 4:**
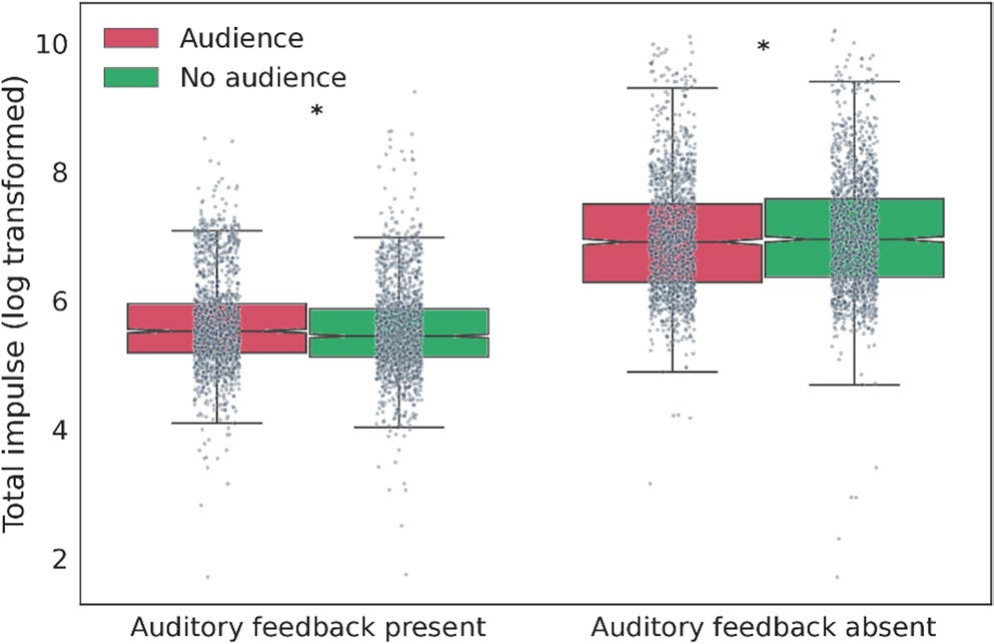
The *Auditory feedback* by *Observation* interaction effect on the Total impulse exerted over the duration of the action (log-transformed). In addition to individual data points, the boxplots display the medians (central line), inter-quartile range (IQR, represented by the box), and outliers (individual points outside the whiskers). The whiskers cover data points up to 1.5 times the IQR from the first and third quartiles. Asterisks mark statistically significant differences. The presence of auditory feedback generally resulted in lower force. However, when auditory feedback was absent, the presence of the observer led to a decrease in force whereas when auditory feedback was present, the presence of the observer led to an increase in force

The total force exerted over the duration of the action (Total impulse) was significantly affected by both the Auditory feedback (χ^2^(1) = 12685.23, *p* < 0.001) and the Order of visit (χ^2^(1) = 77.50, *p* < 0.001). Specifically, the exerted force was lower in the presence of auditory feedback as well as during the second visit (Fig S1B). Note, however, that there were no main effects of Substance administration and Observation on Total impulse (χ^2^(1) = 1.28 and χ^2^(1) = 4.43 respectively, *p* > 0.002 for both). A higher STAI-S score, indicating increased state anxiety, was associated with a higher Total impulse (χ^2^(1) = 99.53, *p* < 0.001) (Fig S2B). In addition, there was a significant interaction between Observation and Auditory feedback (χ^2^(1) = 42.01, *p* < 0.001; Fig. 4). Specifically, when auditory feedback was available, the experimenter’s presence led to stronger force application than her absence, whereas in the absence of auditory feedback, force application was stronger in the experimenter’s absence. Pairwise comparisons revealed a significant difference between the trials with an audience and those without an audience, both when auditory feedback was absent (estimate = 0.05, z = 3.08, *p* = 0.010) and when it was present (estimate = -0.10, z = -6.08, *p* < 0.001).

### Time intervals (T-INT)

**Fig. 5:**
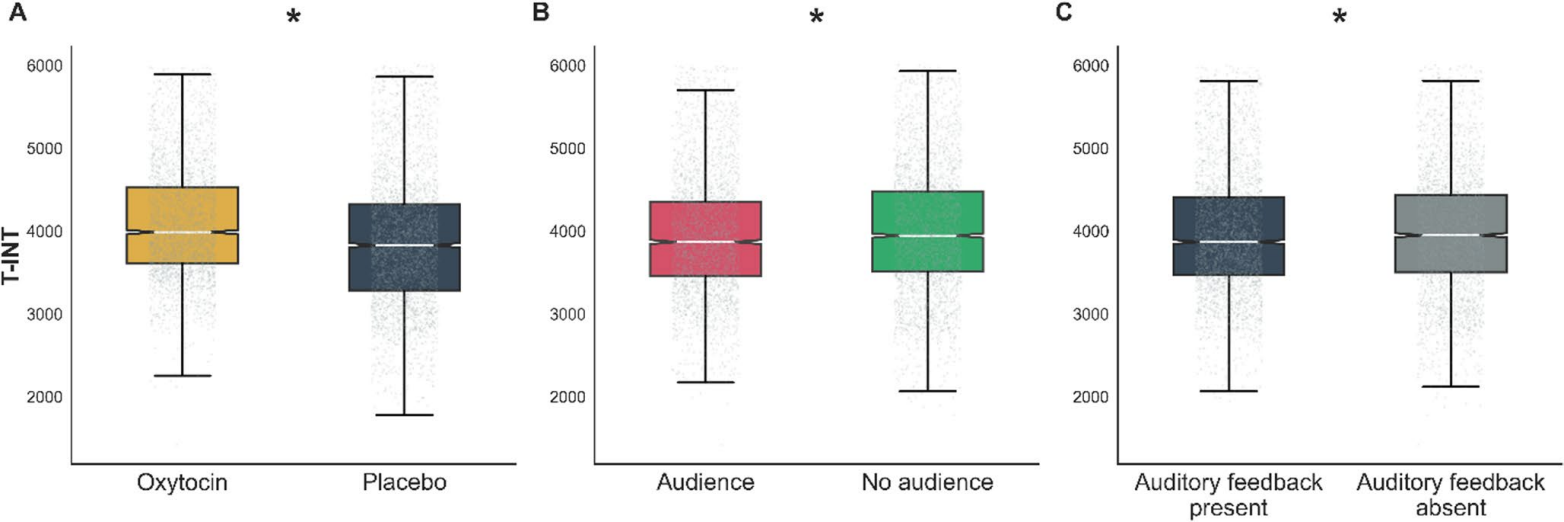
**A** The effect of the *Substance administration* (OXT vs. Placebo) on the trial-level time intervals (T-INT). **B** The effect of the *Observation* on the time intervals (T-INT). **C** The effect of *Auditory feedback* on the time intervals (T-INT). In addition to individual data points, the boxplots display the medians (central line), inter-quartile range (IQR, represented by the box), and outliers (individual points outside the whiskers). The whiskers cover data points up to 1.5 times the IQR from the first and third quartiles. Asterisks mark significant differences

Participants complied with the instructions: the between-action intervals were close to the target 4 s (Fig. 5) (Mean = 3975.2 ms; SD = 752.5 ms). The administration of OXT was associated with prolonged duration between consecutive actions (main effect of Substance administration: χ^2^(1) = 177.25, *p* < 0.001, Fig. 5A). Conversely, being observed by the experimenter and the presence of auditory feedback both resulted in significant reduction in the time intervals (main effect of Observation: χ^2^(1) = 14.05, *p* < 0.001, Fig. 5B; main effect of Auditory feedback: (χ^2^(1) = 12.31, *p* < 0.001, Fig. 5C). When participants returned for their second visit, they exhibited longer T-INT (main effect of Order: χ^2^(1) = 183.68, *p* < 0.001, Fig S1C). The time of day, specifically differences between morning versus mid-day and evening versus mid-day, were significantly related to the T-INT (χ^2^(1) = 33.52, *p* < 0.001), with the longest T-INT observed in the evening, followed by the morning, and then mid-day. (Fig. S3). Furthermore, higher scores on the STAI-S, indicative of increased state anxiety, were also associated with prolonged T-INT (χ^2^(1) = 263.49, *p* < 0.001) (Fig S2C). There were no significant interaction effects. All omnibus tests of main effects and interaction terms, including non-significant results, are reported in Tables S4, S5, S6, S7 and S8.

## Discussion

The current study investigated the effects of oxytocin administration and presence of a human observer on participants’ performance in a simple timing task. Beside the task-relevant between-action interval, we analyzed two metrics reflecting different aspects of force application: whereas Total impulse captured the overall result of both motor planning and potential online force adjustments, Initial impulse captured differences in motor planning affecting only the first 60 ms of the actions. As expected (e.g., Neszmélyi & Horváth, 2017; Horváth et al., 2018), participants responded to the absence of auditory feedback by applying more force, suggesting compensation for increased uncertainty regarding the success of the action. Importantly, the presence of the experimenter led to a reduction of both force measures in the absence of sound feedback, which is compatible with an interpretation that her presence and absence of corrective behavior on her part assured participants about the task-appropriateness of their actions. Significant interactions showed for both measures that this modulation was reduced when sound feedback was present. Although one may suggest that these interactions reflect that the reliable (“objective”) auditory feedback made the reassurance provided by the experimenter’s presence irrelevant, the interpretation is not straightforward, because the modulation of the Initial impulse was reduced, however, the modulation of the Total impulse was actually *reversed*: that is, total impulse increased with auditory feedback when the experimenter was present. At this point, we can only speculate for the reason of this difference between the two measures. As described above, total impulse reflects potential contributions from online adjustments to the actions (Cao et al., 2020; Varga et al., 2022). Typically, such adjustments result in an earlier release of the pressure on the FSR: when hearing the elicited sound, the participant responds by releasing pressure, which leads to shorter and less forceful actions. In the present case, one speculation is that in the presence of the experimenter, participants do not perform such an online adjustment, because they might still be inclined to demonstrate compliance with the task, instead of optimizing their effort by preparing a response to the sound onset. A resolution of this question requires further, more targeted research.

Oxytocin administration resulted in the pronounced reduction of the initial impulse in the presence of a human observer, which indicates that the effect of oxytocin is contingent upon the presence of a human observer. The effect of oxytocin on social behaviors, such as trust and emotion recognition (Van Ijzendoorn & Bakermans-Kranenburg, 2012, Marsh et al., 2010) is well-documented and the studies on oxytocin have largely centered around its role in social bonding (Di Simplicio et al., 2009; Ditzen et al., 2009; Keech et al., 2018). The present finding supports the notion that OXT might prime individuals to be more attuned to social feedback cues. This idea aligns closely with the well-documented ‘audience effect,’ where individuals adjust their behavior when they know they are being observed (Guerin, 1986) and is consistent with oxytocin’s recognized role in social cognition (Hammock, 2015). Although the absence of statistical significance is no evidence for the absence of an effect, the absence of a similar differential Substance administration response for the nonsocial, auditory feedback is compatible with a heightened sensitivity for *social* feedback among participants administered oxytocin. Given the current sample size and the concerns related to the relatively low power of studies on the effects of oxytocin (Walum et al., 2016), the strength of evidence for a null effect in this aspect of the results needs to be assessed in future studies with larger sample sizes.

We found that participants pressed the device at shorter intervals when being watched by an observer, indicating a perception of time passing more quickly in socially intense situations, likely due to the arousing effect of social presence. This finding aligns with existing literature, which suggests that social interactions can compress subjective time perception (Liu et al., 2018). Interestingly, after having received OXT treatment, participants displayed prolonged between-action intervals, which suggest that the time from the previous action was perceived as being shorter than in the condition when they were given a placebo. This result contradicts our initial hypothesis that oxytocin would enhance sensitivity to social stimuli and thereby compress subjective time perception. Instead, oxytocin may have a more complex and individualized effect, potentially enhancing participants’ inner motivational states or emotional engagement (Colonnello et al., 2016; Alaerts et al., 2021), leading to an elongation of subjective time perception in less social contexts too. It appears that oxytocin had an effect in the same direction as anxiety, as measured by the State-Trait Anxiety Inventory (STAI). We acknowledge that oxytocin is often associated with anxiolytic effects (MacDonald & Feifel, 2014; Yoon & Kim, 2022; Bernhard et al., 2018). However, the literature shows a more complex picture (Yoon & Kim, 2022), with both positive (Schuh-Hofer et al., 2018; Saxbe et al., 2019; Lawson et al., 2013; Hoge et al., 2008) and negative correlations (Bernhard et al., 2018; Opacka-Juffry & Mohiyeddini, 2012) reported between oxytocin levels and anxiety, depending on factors such as sex, age, and context (Yoon & Kim, 2022). We believe that oxytocin may enhance the internal motivational states or emotional arousal linked to anxiety, leading to an elongation of subjective time perception. Although the exact mechanism is unclear, it’s possible that oxytocin may either directly influence internal timing mechanisms (Colonnello et al., 2016) or indirectly affect time perception through heightened attention or increased cognitive processing (Xin et al., 2021). The interaction of oxytocin with other neurotransmitters (Shamay-Tsoory & Abu-Akel, 2016), such as dopamine (Love, 2014), which is known to play a role in time perception (Marinho et al., 2018; Fung et al., 2021), could also be a contributing factor. The specific context of the task, participant characteristics, and oxytocin administration details should be considered when interpreting these results. In our study, oxytocin administration led participants to estimate longer time intervals both in a social context (being observed by an experimenter) and in solitude, expanding our understanding of oxytocin’s effects beyond merely social situations.

Heightened state anxiety levels, as measured by the STAI-S affected all the metrics: both force measure levels (Initial impulse and Total impulse) were higher, and between-action intervals were longer. This aligns with the literature suggesting that anxiety can significantly impact motor behavior. Anxiety can affect cognitive control (Aarts & Pourtois, 2010), specifically it increases the sensitivity to errors (Aarts & Pourtois, 2010; Olvet & Hajcak, 2008; Hajcak et al., 2003), which heightens cognitive load by requiring more mental resources for monitoring and correcting performance (Eysenck et al., 2007). The Attentional Control Theory (ACT) proposes that anxiety impairs the goal-directed attentional system while increasing reliance on the stimulus-driven system (Eysenck et al., 2007). This theory distinguishes between performance effectiveness (quality of performance) and processing efficiency (relationship between performance and resource use), with anxiety impairing efficiency more than effectiveness (Derakshan & Eysenck, 2009). Research demonstrates that anxiety reduces attentional control through impaired inhibition and shifting functions (Eysenck et al., 2007; Derakshan & Eysenck, 2009). The increased cognitive effort can disrupt motor control (Nieuwenhuys & Oudejans, 2017), contributing to the observed changes in motor performance and disrupt timing (Matthews & Meck, 2016; Brown, 2008). Specifically high anxiety leads to slower reaction times and compromised motor efficiency, particularly in tasks requiring greater attentional resources (Coombes et al., 2009). Participants may allocate limited processing capacity toward “nontemporal” information at the expense of “temporal” ones, leading to timing inaccuracies (see Brown, 2008, for review). Sport psychology studies show that anxiety reduces the quiet eye period in basketball free throws, supporting ACT’s predictions about impaired goal-directed control (Wilson et al., 2009). Tennis anticipation research reveals anxiety particularly affects high-level cognitive processes and contextual information processing (Cocks et al., 2016). Additionally, another possible explanation for these findings is the role of the body’s physiological response to anxiety, which includes heightened arousal and increased muscle tension (Noteboom et al., 2001; Van Galen et al., 2002; Hazlett et al., 1994). This heightened arousal can lead to changes in motor behavior, including increased force production and altered chronoception (i.e. an elongated between-action interval) (Wittmann, 2015; Hawkes et al., 1962; Van Galen et al., 2002).

### Limitations

Although eye contact was not systematically manipulated, the experimenter’s visual attention likely contributed to the subjective experience of being observed, consistent with prior work showing that gaze cues enhance social salience and evaluative pressure during task performance (Guerin, 1986; Itier & Batty, 2009; Belletier et al., 2015; Cañigueral & Hamilton, 2019). Whereas the within-subject design and the task paradigm is well-powered to detect within-person differences in motor adaptation, the relatively small sample size (*N* = 18) limits the generalizability of some of the findings, particularly in the context of oxytocin research where effect sizes are often modest and subject to variability. The small sample size also increases the possibility of both Type II errors and inflated effect-size estimates, therefore these findings warrant replication in larger, more diverse samples. Additionally, the study included only male participants to reduce variability linked to menstrual cycle and hormonal fluctuations; while this is a common practice in oxytocin research, it limits clinical applicability, as effects observed in males may not generalize to females. Importantly, the observation condition involved a female experimenter and male participants, resulting in a cross-gender social context. Social evaluative effects are known to be sensitive to characteristics of both the observer and the observed individual. Prior research suggests that cross-gender settings can heighten self-presentational and performance-related processes (e.g., Castrellon et al., 2024; Karremans et al., 2009), and that characteristics such as perceived status or age of the evaluator may modulate responses to social pressure. Because experimenter sex, age, and perceived authority were not systematically manipulated or assessed, we cannot disentangle general audience effects from potential cross-gender or authority-related influences. Although most participants were in their twenties, the inclusion of a wider age range (20–50 years) may have increased inter-individual variability in timing behavior. Further studies are needed to determine whether the present effects generalize across different experimenter-participant gender and age constellations.

## Conclusions

In conclusion, the results of the present study support the role of oxytocin in optimizing motor performance and timing in contexts that have high social relevance. Its underlying mechanisms might be anchored in social sensitivity leading to an increased sensitivity to observation as well, and it might have a broader impact on internal timing mechanisms and cognitive processes, within and beyond social interactions. Collectively, the findings underscore the intricate balance between factors like oxytocin, observation, auditory feedback, and participant anxiety in modulating performance metrics in the timing task.

## Supplementary Information

Below is the link to the electronic supplementary material.

Supplementary Material 1.

## Data Availability

The data that support the findings of this study are available from the corresponding author, JH, upon reasonable request.
