# Supplementary material for: The impact of intranasal oxytocin administration and social observation on time perception and task execution in a simple motor task
Source: Psychol Res. 2026 Apr 7;90(2):68. doi: 10.1007/s00426-026-02290-w (PMC13056776; doi:10.1007/s00426-026-02290-w)
Supplement: Supplementary file 1 — Supplementary Material 1. [file 426_2026_2290_MOESM1_ESM.docx]

**Supplementary Material**

**The impact of intranasal oxytocin administration and social observation on time perception and task execution in a simple motor task**

Orsolya Kiss, József Topál, Dorottya Berkes, Karolin Török-Suri, János Horváth

**Supplementary Table S1.** Correlation metrics for motor task outcome measures. This figure displays the Pearson correlation matrix for all the motor task metrics, highlighting the pairwise relationships between different outcome measures. Each cell in the matrix represents the correlation coefficient between two motor metrics

| Variables | PL | Total impulse | Initial impulse | | DA | T-INT |
| --- | --- | --- | --- | --- | --- | --- |
| PF | 0.46 | 0.86 | 0.49 | 0.47 | | 0.11 |
| PL |  | 0.66 | 0.14 | 0.93 | | 0.07 |
| Total impulse |  |  | 0.37 | 0.72 | | 0.14 |
| Initial impulse |  |  |  | 0.21 | | 0.11 |
| DA |  |  |  |  | | 0.06 |

**Supplementary Table S2.** Linear mixed-effects model output for the analysis of the Total impulse (log-transformed), the Initial impulse (log-transformed) and the time intervals between the consecutive actions (T-INT).

| **Performance measures** | **Total impulse** | | | | **Initial impulse** | | | | **T-INT** | | | |
| --- | --- | --- | --- | --- | --- | --- | --- | --- | --- | --- | --- | --- |
| Predictors | χ2 (1) | *p* | | χ2 (1) | | *p* | | χ2 (1) | | *p* | |  |
| Substance condition (OXT vs placebo) | 1.28 | | 0.258 | | 28.49 | | <0.001 | | 177.25 | | <0.001 | |
| Observation (audience vs non audience) | 4.43 | | 0.035 | | 196.01 | | <0.001 | | 14.05 | | <0.001 | |
| Auditory feedback (yes/no) | 12685.23 | | <0.001 | | 6615.03 | | <0.001 | | 12.31 | | <0.001 | |
|  |  | |  | |  | |  | |  | |  | |
| STAI State | 99.53 | | <0.001 | | 67.86 | | <0.001 | | 264.69 | | <0.001 | |
| Order of the visit | 77.50 | | <0.001 | | 35.64 | | <0.001 | | 183.68 | | <0.001 | |
| Time of day | - | | - | | - | | - | | 33.52 | | <0.001 | |
| Substance condition x Observation | 1.02 | | 0.313 | | 14.06 | | <0.001 | | 0.62 | | 0.432 | |
| Substance condition by Auditory feedback | 0.93 | | 0.335 | | 0.01 | | 0.940 | | 0.09 | | 0.769 | |
| Observation by Auditory feedback | 42.01 | | <0.001 | | 41.80 | | <0.001 | | 0.86 | | 0.355 | |
|  |  | |  | |  | |  | |  | |  | |

**Note**: The STAI scores were standardized. The random-effects structure included a random intercept for participant ID, and a random slope for the trial order.

**Supplementary Table S3.** Fixed effect estimates from the linear mixed-effects model predicting Initial impulse (log-transformed). The table reports unstandardized estimates, standard errors, t-values, and p-values for each predictor, including main effects and interaction terms.

| **Performance measures** | **Initial impulse** | | | |
| --- | --- | --- | --- | --- |
| Predictors | Estimate | Std. error | *t value* | *p* |
| Intercept | 4.99 | 0.02 | 200.80 | <0.001 |
| Substance condition - OXT vs placebo (ref) | -0.01 | 0.01 | -1.10 | 0.271 |
| Observation (audience vs non audience) | -0.05 | 0.01 | -9.85 | <0.001 |
| Auditory feedback (yes vs no) | -0.28 | 0.01 | -51.56 | <0.001 |
| STAI State | 0.04 | 0.00 | 8.24 | <0.001 |
| Order of the visit | -0.02 | 0.00 | -5.97 | <0.001 |
| Substance condition x Observation | -0.02 | 0.01 | -3.75 | <0.001 |
| Substance condition by Auditory feedback | 0.00 | 0.01 | 0.07 | 0.94 |
| Observation by Auditory feedback | 0.04 | 0.01 | 6.47 | <0.001 |
|  |  |  |  |  |

**Note**: The STAI scores were standardized. The random-effects structure included a random intercept for participant ID, and a random slope for the trial order. Analysis included 6659 data points across 18 participants. The model accounted for 64% of the Initial impulse data (adjusted pseudo-*R*^2^ = .64).

**Supplementary Table S4.** Fixed effect estimates from the linear mixed-effects model predicting Total impulse (log-transformed). The table reports unstandardized estimates, standard errors, t-values, and p-values for each predictor, including main effects and interaction terms.

| **Performance measures** | **Total impulse** | | | |
| --- | --- | --- | --- | --- |
| Predictors | Estimate | Std. error | *t value* | *p* |
| Intercept | 7.10 | 0.13 | 55.06 | <0.001 |
| Substance condition - OXT vs placebo (ref) | -0.04 | 0.02 | -1.79 | 0.073 |
| Observation (audience vs non audience) | -0.07 | 0.02 | -3.14 | 0.002 |
| Auditory feedback (yes vs no) | -1.52 | 0.02 | -70.46 | <0.001 |
| STAI State | 0.17 | 0.02 | 9.98 | <0.001 |
| Order of the visit | -0.13 | 0.01 | -8.80 | <0.001 |
| Substance condition x Observation | 0.03 | 0.03 | 1.01 | 0.313 |
| Substance condition by Auditory feedback | 0.02 | 0.03 | 0.96 | 0.335 |
| Observation by Auditory feedback | 0.16 | 0.03 | 6.48 | <0.001 |
|  |  |  |  |  |

**Note**: The STAI scores were standardized. The random-effects structure included a random intercept for participant ID, and a random slope for the trial order. Analysis included 6659 data points across 18 participants. The model accounted for 78% of the variance in the Total impulse data (adjusted pseudo-*R*^2^ = .78).

**Supplementary Table S5.** Fixed effect estimates from the linear mixed-effects model predicting **T-INT** (time intervals between actions). The table reports unstandardized estimates, standard errors, t-values, and p-values for each predictor, including main effects and interaction terms.

| **Performance measures** | **T-INT** | | | |
| --- | --- | --- | --- | --- |
| Predictors | Estimate | Std. error | *t value* | *p* |
| Intercept | 3950.72 | 164.93 | 23.95 | <0.001 |
| Substance condition - OXT vs placebo (ref) | 140.12 | 18.93 | 7.40 | <0.001 |
| Observation (audience vs non audience) | -38.32 | 18.45 | -2.08 | 0.038 |
| Auditory feedback (yes vs no) | -31.09 | 18.20 | -1.71 | 0.088 |
| STAI State | 300.85 | 18.49 | 16.27 | <0.001 |
| Order of the visit | 172.37 | 12.72 | 13.55 | <0.001 |
| Time of day (<12 PM) | 102.28 | 38.35 | 2.67 | 0.008 |
| Time of day (>4 PM) | 115.28 | 20.25 | 5.69 | <0.001 |
| Substance condition x Observation | 16.94 | 21.56 | 0.79 | 0.432 |
| Substance condition by Auditory feedback | 6.36 | 21.63 | 0.29 | 0.769 |
| Observation by Auditory feedback | -19.95 | 21.55 | -0.93 | 0.355 |
|  |  |  |  |  |

**Note**: The STAI scores were standardized. The random-effects structure included a random intercept for participant ID, and a random slope for the trial order. Analysis included 6088 data points across 18 participants. The model accounted for 78% of the variance in the T-INT data (adjusted pseudo-*R*^2^ = .78).

**Supplementary Table S6.** Post-hoc pairwise comparisons – Initial Impulse: Substance condition x Observation

| Contrast | Estimate | SE | t | p (adj.) |
| --- | --- | --- | --- | --- |
| placebo, non audience – OXT, non audience | 0.0059 | 0.0046 | 1.29 | .568 |
| placebo, non audience – placebo, audience | 0.0333 | 0.0044 | 7.51 | <.001 |
| placebo, non audience – OXT, audience | 0.0632 | 0.0046 | 13.75 | <.001 |
| OXT, non audience – Placebo, audience | 0.0274 | 0.0046 | 5.95 | <.001 |
| OXT, non audience – OXT, audience | 0.0573 | 0.0046 | 12.45 | <.001 |
| placebo, audience – OXT, audience | 0.0298 | 0.0046 | 6.44 | <.001 |

Note: We report Tukey-adjusted p-values.

**Supplementary Table S7.** Post-hoc pairwise comparisons – Initial Impulse: Auditory feedback x Observation

| **Contrast** | **Estimate** | **SE** | **t** | **p (adj.)** |
| --- | --- | --- | --- | --- |
| no feedback, non audience – feedback, non audience | 0.2802 | 0.0045 | 62.44 | <.001 |
| no feedback, non audience – no feedback, audience | 0.0659 | 0.0045 | 14.55 | <.001 |
| no feedback, non audience – feedback, audience | 0.3049 | 0.0045 | 67.47 | <.001 |
| feedback, non audience – no feedback, audience | −0.2143 | 0.0045 | −47.37 | <.001 |
| feedback, non audience – feedback, audience | 0.0246 | 0.0045 | 5.47 | <.001 |
| no feedback, audience – feedback, audience | 0.2389 | 0.0046 | 52.48 | <.001 |

Note: We report Tukey-adjusted p-values.

**Supplementary Table S8.** Post-hoc pairwise comparisons – Total Impulse: Auditory feedback x Observation

| **Contrast** | **Estimate** | **SE** | **t** | **p (adj.)** |
| --- | --- | --- | --- | --- |
| no feedback, non audience – feedback, non audience | 1.5065 | 0.0178 | 84.71 | <.001 |
| no feedback, non audience – no feedback, audience | 0.0555 | 0.0180 | 3.09 | .011 |
| no feedback, non audience – feedback, audience | 1.3979 | 0.0179 | 78.07 | <.001 |
| feedback, non audience – no feedback, audience | −1.4511 | 0.0179 | −80.94 | <.001 |
| feedback, non audience – feedback, audience | −0.1087 | 0.0179 | −6.09 | <.001 |
| no feedback, audience – feedback, audience | 1.3424 | 0.0180 | 74.42 | <.001 |

Note: We report Tukey-adjusted p-values.

**Supplementary Table S9.** Model Comparison Across Upper-Interval Thresholds

| Predictor | Original (6s)  LR χ² (p-value) | 7 s  LR χ² (p-value) | 8s  LR χ² (p-value) | No Upper Limit  LR χ² (p-value) |
| --- | --- | --- | --- | --- |
| Substance condition | 177.25 (<.001) | 219.19 (<.001) | 270.31 (<.001) | 272.29 (<.001) |
| Observation | 14.05 (<.001) | 10.84 (.001) | 3.88 (.049) | 1.36 (.243) |
| Auditory Feedback | 12.31 (<.001) | 7.59 (.006) | 6.41 (.011) | 5.75 (.016) |
| STAI (state) | 264.69 (<.001) | 335.62 (<.001) | 279.91 (<.001) | 216.15 (<.001) |
| Order | 183.68 (<.001) | 291.72 (<.001) | 357.21 (<.001) | 343.14 (<.001) |
| Daytime | 33.52 (<.001) | 102.35 (<.001) | 246.02 (<.001) | 352.76 (<.001) |
| Substance condition × Observation | 0.62 (.432) | 2.26 (.132) | 1.54 (.215) | 1.24 (.266) |
| Substance condition × Auditory Feedback | 0.09 (.769) | 4.12 (.042) | 5.12 (.024) | 4.04 (.044) |
| Observation × Feedback | 0.86 (.355) | 0.04 (.836) | 0.26 (.607) | 0.70 (.404) |

Note: Values represent likelihood-ratio χ² statistics with associated, non-adjusted p-values.


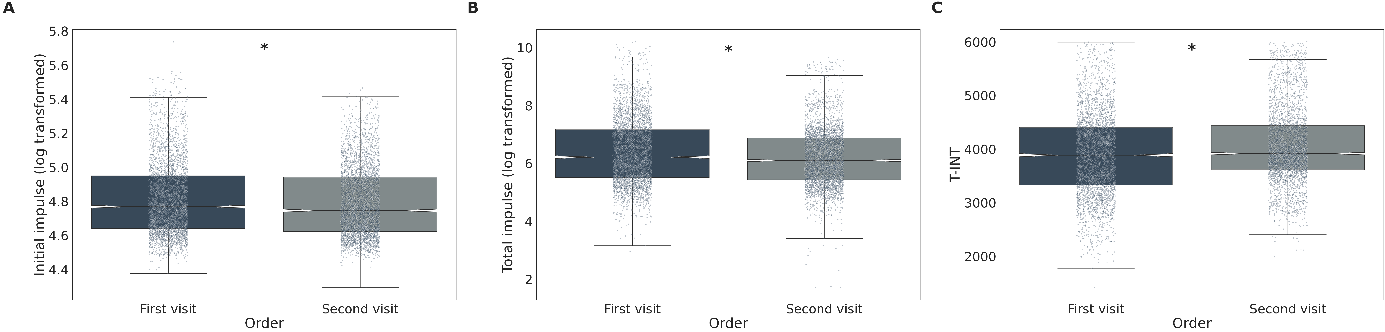


**Figure S1**: The effect of the visit order on the Initial impulse, Total impulse, and T-INT, and the time intervals between the consecutive actions – T-INT. In addition to individual data points, the boxplots display the medians (central line), inter-quartile range (IQR, represented by the box), and outliers (individual points outside the whiskers). The whiskers cover datapoints up to 1.5 times the IQR from the first and third quartiles. Asterisks mark significant differences


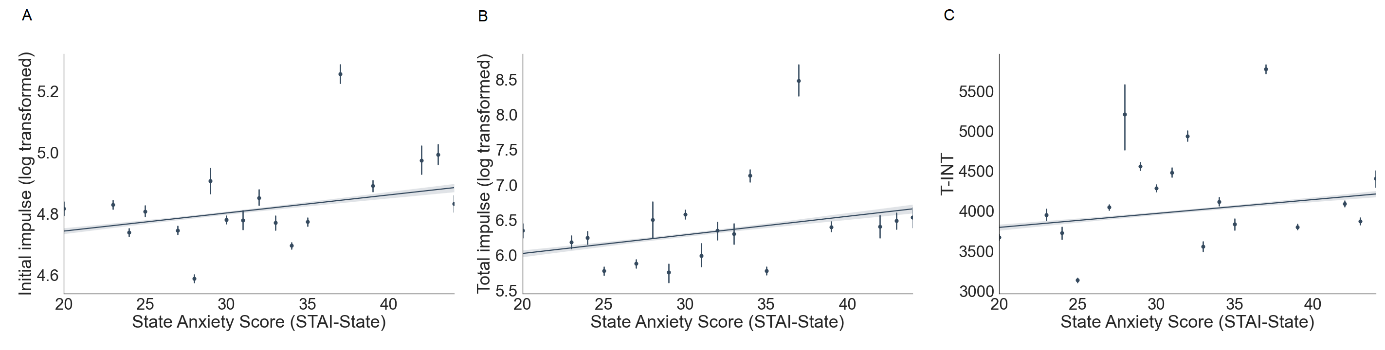


**Figure S2:** The association between State Anxiety, measured by the State – Trait Anxiety Inventory and the Initial impulse, Total impulse, and the time intervals between the consecutive actions – T-INT (dots: mean ± CI). We provide trend line fitted using linear regression.


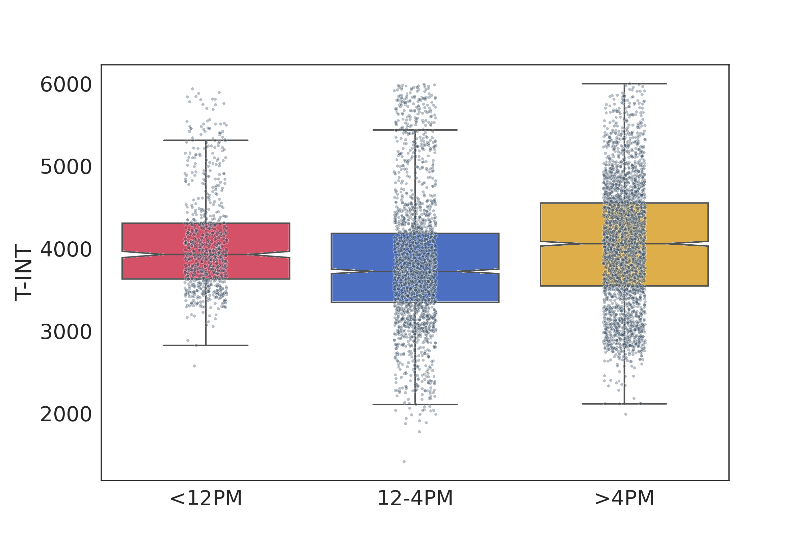


**Figure S3**: The effect of the time of day (medians ± IQT and outliers) on the time intervals (T-INT). In addition to individual data points, the boxplots display the medians (central line), inter-quartile range (IQR, represented by the box), and outliers (individual points outside the whiskers). The whiskers cover data points up to 1.5 times the IQR from the first and third quartiles.
